# Antifungal susceptibility and genomic profile associated with biosynthetic gene clusters of bovine mastitis-derived *Aspergillus fumigatus* DAH1043FM

**DOI:** 10.3389/fmicb.2026.1928624

**Published:** 2026-09-30

**Authors:** Abhishek Prajapati, Pooja Parmar, Monica Chavan, Vartika Chandra, Satyamitra Shekh, Ashish Patel, Pritesh Sabara, Amit Kanani, Snehal Bagatharia, Chaitanya Kumar Jha

**Affiliations:** 1 Department of Microbiology and Biotechnology, University School of Sciences, Gujarat University, Ahmedabad, Gujarat, India; 2 Department of Microbiology, Gujarat Arts and Science College, Ahmedabad, Gujarat, India; 3 Office of Deputy Director of Animal Husbandry, FMD Typing Scheme, Pashupalan Sankul, Ahmedabad, Gujarat, India; 4 Gujarat Biotechnology Research Center, Department of Science and Technology, Gandhinagar, Gujarat, India

**Keywords:** comparative genomics, echinocandins, fungal pathogen, intramammary infection, Oxford Nanopore Technologies, secondary metabolites, whole-genome sequencing

## Abstract

The most frequently isolated fungus from mycotic mastitis in cattle is *Aspergillus fumigatus*, which can cause infection by germinating on the animal's surface to form hyphae that penetrate epithelial and endothelial barriers and initiate mastitis. In this study, the fungal strain DAH1043FM was isolated from the milk of a cow with mastitis and identified as *Aspergillus fumigatus* by internal transcribed spacer (ITS) region sequencing. Species identity was further confirmed by whole-genome Average Nucleotide Identity (ANI) analysis. *A. fumigatus* DAH1043FM showed minimum inhibitory concentration (MIC) values of 0.5 μg/mL for amphotericin B and itraconazole, 0.12 μg/mL for posaconazole and voriconazole, 128 μg/mL for fluconazole, and >8 μg/mL for caspofungin, micafungin, and anidulafungin. Comparative Whole-genome sequencing (WGS) with two reference genomes of *A. fumigatus*, Af293 and A1163, was used to identify genetic variants, such as single nucleotide polymorphisms (SNPs) and other mutations, in genes associated with antifungal resistance, including those involved in drug efflux, ergosterol biosynthesis, echinocandin target pathways, and other resistance-related processes. Furthermore, genome mining identified biosynthetic gene clusters (BGCs) showing substantial similarity to characterized pathways associated with fumiquinazoline (100%), gliotoxin (84%), fumagillin (80%), and trypacidin (71%). These results demonstrate that strain DAH1043FM carries predicted genetic variants in target pathways while exhibiting wild-type susceptibility to azoles alongside off-scale endpoints for echinocandins. Genome analysis of strain DAH1043FM identified predicted secondary metabolite BGCs, highlighting the value of genome-level surveillance for fungal isolates recovered from bovine milk.

## Introduction

There are several species of *Aspergillus* fungi, and many of these are reported to cause diseases in dairy animals, especially mastitis. Notably, *A. flavus, A. niger, A. nidulans*, and *A. terreus* have been reported more frequently in bovine mycotic mastitis cases (Pal, 2023; Belete Temesgen et al., 2026). Another thermotolerant fungus found in soil, decomposing plant material, and spoilt cattle feed, *Aspergillus fumigatus*, is also one of the major fungal pathogens in mastitis in cattle (Kot et al., 2026). *A. fumigatus* is a pathogenic fungus that causes various diseases in dairy cattle, such as bovine abortion and reproductive disorders (Pal et al., 2024), bovine mastitis (Belete Temesgen et al., 2026), and bovine respiratory diseases (Centeno-Martinez et al., 2023). *A. fumigatus* produces several secondary metabolites that are highly harmful to animal and human health, such as fumagillin, fumitoxins, fumigaclavines A and C, fumitremorgins, gliotoxins, and fumiquinazolines (Mohamed et al., 2023). Several classes of antifungal drugs are available; however, azoles, polyenes, and echinocandins are the primary classes used against *Aspergillus* infections, and azoles are the first drug of choice. However, azole drug efficacy is threatened by the worldwide spread of resistance (Garcia-Rubio et al., 2018).

Mycotic mastitis associated with *Aspergillus* species in dairy cattle is more frequently reported and can result in significant economic losses due to decreased milk production, failed treatment attempts, and culling in severe cases (Sharun et al., 2021; Corrêa et al., 2024). *A. fumigatus* is one of the filamentous fungi identified to be associated with bovine mastitis (Ahmed et al., 2024). However, most studies have focused on conventional culture and molecular identification methods (Alshehri and Palanisamy, 2020; Ahmed et al., 2024), whereas only a few studies have been performed on genome-scale characterization of the fungus (Barber et al., 2021). In addition, the genomic distribution of biosynthetic gene clusters (BGCs), which encode the enzymatic machinery for synthesizing secondary metabolites such as mycotoxins, remains poorly defined in *Aspergillus fumigatus* isolates originating from bovine mastitis. While specific secondary metabolites are established virulence factors that promote fungal survival and host tissue damage, the genomic potential and expression of these multigene pathways in intramammary infection remain understudied (Steenwyk et al., 2020; Seo et al., 2024). This is a major knowledge gap, with the absence of more extensive genomic data on bovine mastitis-associated *A. fumigatus*, which would facilitate understanding of the genetic diversity, antifungal resistance mechanisms, and toxigenic potential of this fungus.

*Aspergillus fumigatus* is genetically diverse, with lineage-specific variations and differences in antifungal resistance genes identified among its populations. Therefore, whole-genome approaches are critical for understanding strain-level adaptation and pathogenicity (Barber et al., 2021; Lofgren et al., 2022). Similarly, the global occurrence of azole-resistant *A. fumigatus* has become a significant issue, in which resistance is often associated with mutations in the *cyp51A* gene, as well as in genes associated with ergosterol biosynthesis, stress response, and drug efflux mechanisms (Verweij et al., 2015; Hosseini et al., 2024; Onchan et al., 2025; Fengler et al., 2026). *A. fumigatus*, identified as a fungal pathogen, is now a global issue due to the emergence of resistance; hence, the World Health Organization (WHO) added this pathogen to the fungal priority pathogen list (World Health Organization, 2022). Although much is known about the mechanisms of antifungal resistance in *A. fumigatus*, this knowledge is derived largely from human clinical and environmental isolates (Puértolas-Balint et al., 2019; Barber et al., 2021; Rhodes et al., 2022). However, human clinical findings cannot be directly applied to bovine mastitis strains because dairy cattle have different physiological conditions, immune defenses, and specific treatment exposures. In contrast, genomic information on bovine mastitis-associated fungi is still lacking, particularly regarding resistance-associated mutations and virulence-associated genomic characteristics.

The present study aims to assess the susceptibility profile and virulence potential of *A. fumigatus* pathogen involved in bovine mastitis. We hypothesized that the mastitis-derived *A. fumigatus* strain possesses specific genomic variations in antifungal resistance-associated genes and encodes intact BGCs capable of producing mycotoxins that contribute to its pathogenic potential. To test this hypothesis, the objectives of the study are to examine the dairy cow for mastitis using the California Mastitis Test (CMT) and isolate mastitis-associated fungus; identify the fungal pathogen using a molecular approach involving sequencing of the ITS region; evaluate its antifungal susceptibility profile and whole-genome sequencing (WGS)-based characterization of antifungal resistance genes and associated mutations; and identify predicted secondary metabolites BGCs using *in silico* analysis to evaluate the genomic potential for toxin biosynthesis in fungus isolated from bovine milk.

## Materials and methods

### Sample collection and detection of invasive fungal infection

A milk sample was collected from a lactating cow in late lactation with recurrent subclinical mastitis that had not responded to previous antibacterial treatment. The sample was collected from a dairy farm in Ahmedabad, India (22.8825° N, 72.5633° E), following the necessary guidelines (Lima et al., 2018) and was tested using the CMT (Alkhouly et al., 2023). The first few streams of milk from each quarter were discarded to stimulate mammary gland function, and the milk sample was collected aseptically. The teats were cleaned and disinfected using 70% ethanol, and the milk sample was collected in a sterile container without preservatives and transported to the laboratory at 4 °C within 2 h (National Mastitis Council Procedures for Collecting Milk Samples, 2004).

Fungal DNA extraction was performed from milk by filling 10 mL of milk into a sterile 15 mL conical tube, which was centrifuged at 6,000 x g for 15 min at 4 °C. After carefully removing the upper layer of fat, the pellet was given two sterile Phosphate Buffer Solution (PBS) washes. During each wash, the suspension was centrifuged for 5 min at 10,000 x g, and the supernatant was discarded (Liu et al., 2014; Volk et al., 2014). 500 μL of CTAB lysis solution containing 2% CTAB, 100 mM Tris-HCl (pH 8.0), 20 mM EDTA, 1.4 M NaCl, 1% PVP, and 1% SDS was used to lyse the cleaned pellet. After adding 0.3 g of 0.5 mm beads, the sample was bead-beaten twice for 1 min each. The lysate was then treated for 30 min at 56 °C with proteinase K, and the sample was mixed with an equal volume of chloroform: isoamyl alcohol (24:1) and centrifuged for 10 min at 12,000 x g. The aqueous phase was collected in a fresh tube. DNA was precipitated by adding 0.7 volumes of cold isopropanol and incubating at −20 °C for 30 min. The sample was centrifuged at 12,000 x g for 10 min; the supernatant was removed, and the pellet was washed with 70% ethanol. After brief air-drying, DNA was resuspended in 50 μL TE buffer (Dar et al., 2025; Griffiths et al., 2006; Liu et al., 2014; Volk et al., 2014). Polymerase chain reaction (PCR) was performed using ITS1 and ITS4 primers (Center of Expertise in Mycology Radboudumc/CWZ, 2019).

### Isolation and molecular identification of DAH1043FM by ITS amplification

The positive sample was cultured on Sabouraud dextrose agar (SDA) containing 0.05% chloramphenicol (HiMedia Laboratories, India-MAP1067) to inhibit bacterial colonies and incubated at 35 °C for up to seven days (Verdier-Metz et al., 2022). To detect bacterial presence in the milk, parallel bacteriological culturing was performed by plating the pooled milk sample onto 5% Sheep blood agar (HiMedia Laboratories, India-M073) and MacConkey agar (HiMedia Laboratories, India-M008E) and incubated at 37 °C for 24 hours.

DNA extraction from the fungal colony was performed using the HiPurA^®^ Fungal DNA Purification Kit (HiMedia Laboratories, India-MB543) according to the manufacturer's protocol. Qualitative analysis of DNA was performed by 0.8% agarose gel electrophoresis, and a quantitative test was performed using the Cytation 5 multimode reader (Agilent BioTek, USA) and Qubit 4 Fluorometer (Invitrogen™, Thermo Fisher Scientific, USA). PCR was performed using ITS1 (5′-TCCGTAGGTGAACCTGCGG-3′) and ITS4 (5′-TCCTCCGCTTATTGATATGC-3′) primers. Each 20 μL PCR reaction mixture contained 10 μL of 2X Master Mix, 0.5 μL of forward primer (10 pmol), 0.5 μL of reverse primer (10 pmol), 6.5 μL of nuclease-free water (NFW), and 2.5 μL of template DNA. A positive control containing DNA of *Aspergillus fumigatus* ATCC 204305 and a no-template control (NTC) containing NFW were included in the PCR run. Amplification was conducted using the following thermocycling conditions: initial denaturation at 95 °C for 5 min, followed by 40 cycles of denaturation at 94 °C for 1 min, annealing at 55 °C for 30 s, and extension at 72 °C for 1 min, with a final extension step at 72 °C for 7 min, ending with a hold at 4 °C.

Sanger sequencing of the amplified ITS region was performed (Alshehri and Palanisamy, 2020) using the BigDye™ Terminator v3.1 Cycle Sequencing Kit (Applied Biosystems, USA) and the ABI 3100 genetic analyzer (Applied Biosystems, USA). The raw file was curated in Chromas v2.6.6 (Technelysium Pty Ltd., Australia) and subjected to BLASTn (https://blast.ncbi.nlm.nih.gov). NCBI GenBank submission of the ITS sequence was performed. MEGA v12 and iTOL v7 (https://itol.embl.de) were used to generate and edit the phylogenetic tree using the maximum likelihood method (Kumar et al., 2024; Letunic and Bork, 2024).

### Antifungal susceptibility test of *A. fumigatus* DAH1043FM

Minimum inhibitory concentrations (MICs) of the *A. fumigatus* DAH1043FM strain against eight antifungal drugs were determined using the Octofungi 1 HiMIC plate kit (HiMedia Laboratories, India-MPK901) following the manufacturer's protocol for filamentous fungi. Conidia were harvested from 7-day-old culture of *A. fumigatus* DAH1043FM grown on SDA at 35 °C in sterile HiMIC™ diluent tubes. Inoculum density was adjusted spectrophotometrically at 530 nm to an optical density of 0.09–0.13, yielding a final working inoculum of 0.4 x 10^4^ to 5 x 10^4^ CFU/mL. Serial dilutions of antifungal drugs were provided in the Octofungi 1 kit. Dilution ranges were 0.008–8 μg/mL for amphotericin B, echinocandins, posaconazole, and voriconazole; 0.25–256 μg/mL for fluconazole; and 0.016–16 μg/mL for itraconazole. A plate was incubated at 35 °C and visually read after 48 h. The endpoint for antifungal susceptibility was determined colorimetrically in the Octofungi 1 HiMIC system. Fungal growth causes a color change from purple to pink due to metabolic reduction. The MIC was defined as the lowest concentration of the antifungal agent that completely inhibited growth, maintaining the original purple color indicator. Assays were performed in triplicate on three independent experimental days to ensure reproducibility. *Aspergillus fumigatus* ATCC 204305 was included as a standard reference quality control strain to validate testing conditions and drug concentrations.

MIC values for amphotericin B, itraconazole, posaconazole, and voriconazole were interpreted based on the breakpoints established by the Indian Council of Medical Research (ICMR) (Center of Expertise in Mycology Radboudumc/CWZ, 2019). However, no established breakpoints are available for anidulafungin, caspofungin, micafungin, or fluconazole against *A. fumigatus* (Supplementary Table 1).

### WGS analysis

#### Library preparation and genome preprocessing

Genomic DNA was end-repaired, dA-tailed, and barcoded using the Native Barcoding Kit 24 V14 (SQK-NBD114.24, Oxford Nanopore Technologies, UK). WGS was performed using an Oxford Nanopore Technologies (ONT) MinION Mk1B platform using R10.4.1 flow cells. Raw data were base called using Guppy v6.5.7 under the High-Accuracy (HAC) base calling model. Automated demultiplexing was performed directly by the sequencing software based on the specific barcode assignments. Raw ONT reads were subsequently processed for quality control, adapter trimming, and filtering to retain high-quality reads meeting a minimum Phred quality score threshold (Q ≥ 10) and a minimum read length cutoff (> 500 bp) using CLC Genomics Workbench v25.0.2 (QIAGEN, Denmark). *De novo* genome assembly was executed using CLC Genomics Workbench v25.0.2 under default long-read assembly parameters. Subsequent consensus correction was performed using Medaka v1.8.0 for one round of polishing. The assembled genome was quality-checked using QUAST v5.3.0 (https://github.com/ablab/quast) and BUSCO v2.0 to assess genome completeness and screen for potential assembly contamination or lineage anomalies. Finally, the Funannotate pipeline v1.8.17 (https://github.com/nextgenusfs/funannotate) was used for downstream quality curation and preprocessing of the assembled genome (Palmer and Stajich, 2020).

#### Structural gene prediction

Gene models were predicted using both *ab initio* and evidence-based methods (Onchan et al., 2025). DIAMOND and Exonerate were used to align conserved fungal proteins to the genome, which provides protein evidence for predicting gene structure. The *ab initio* gene prediction tools SNAP (Johnson et al., 2008) and GlimmerHMM (Majoros et al., 2004) were trained using BUSCO-derived conserved genes, while Augustus (Stanke et al., 2008) gene predictions were performed using pre-trained *A. fumigatus* parameters. The gene predictions from Augustus, SNAP, and GlimmerHMM were combined with Evidence Modeler (EVM) (Haas et al., 2008) to produce consensus gene models. tRNAscan-SE was used to identify transfer RNA (tRNA) genes, and the final structural annotation set was created for further functional annotation analysis.

#### Functional annotation of predicted genes

Protein domains were found using HMMER searches using the Pfam database (Pei et al., 2024). Functional descriptions and gene names were assigned based on similarity searches performed using DIAMOND against the UniProt database (Ahmad et al., 2025). EggNOG-Mapper (Cantalapiedra et al., 2021) was used to classify orthologous groups, create functional categories, and annotate pathways. Additional protein functional characterization included searches against the MEROPS database (Rawlings et al., 2014) for proteases and the dbCAN database (Yin et al., 2012) for carbohydrate-active enzymes (CAZymes). Conserved fungal orthologs were further identified using the BUSCO dikarya_odb9 lineage dataset. The resulting annotations were integrated to generate a comprehensive functional annotation dataset for all predicted genes.

### Genome-based species confirmation using average nucleotide identity (ANI) analysis

A whole-genome ANI was performed using FastANI v1.3 for genome-based confirmation of the species DAH1043FM (Jain et al., 2018). The assembled genome of DAH1043FM was compared with the reference genome of *A. fumigatus* Af293 (GCA_000002655.1). The genomic similarity between the query and reference genomes was evaluated by calculating the ANI value after FastANI was performed using the default parameters.

### Profiling of AMR genes and mutational analysis

The genes identified in DAH1043FM were compared against the fungal CARD mutational data to identify AMR-associated genes. MEGA v12 was used for multiple sequence alignment (MSA) of extracted common genes (Kumar et al., 2024). Two well-characterized, wild-type susceptible reference genomes, *Aspergillus fumigatus* Af293 and *Aspergillus fumigatus* A1163, were used as baselines for comparative sequence analysis (Garcia-Rubio et al., 2018). Gene sequences exhibiting 100% sequence identity with these susceptible reference genomes were classified as baseline wild-type alleles. Sequences showing nucleotide variations were further evaluated by SNP analysis, amino acid substitution mapping, and truncation assessment to evaluate potential correlations with the observed phenotypic resistance profiles.

To verify that identified mutational features, frame shifts, indels, truncations, and SNPs represented true genomic variations rather than *de novo* assembly or sequencing artifacts, automated read-level variant validation was performed. Filtered ONT long reads were mapped to the final consensus assembly using Minimap2, and the resulting alignment files were evaluated using bcftools v1.13 under a haploid model (–ploidy 1). Variant calls were stringently filtered for high quality (QUAL > 30) and minimum read coverage depth (DP > 20 X). Complete concordance between the aligned read sequences and the consensus assembly across all targeted loci confirmed that the reported structural variations and amino acid modifications represent read-verified genomic traits of strain DAH1043FM.

### Detection of secondary metabolites BGCs

Secondary metabolite BGCs were predicted using antiSMASH v6 (Blin et al., 2021). The predicted BGCs were subsequently classified and compared with known clusters to assess the secondary metabolite biosynthetic potential of the fungal isolate. To ensure high-confidence functional prediction and prioritize pathways with strong evidence for conserved secondary metabolite biosynthesis, a minimum similarity threshold of 70% to known antiSMASH reference clusters was applied.

## Results

### Isolation of fungal strain from mastitis milk

On-farm collection and testing of a milk sample for mastitis was performed using CMT. Individual quarter screening via CMT identified positive reactions in the Right Hind (RH) and Left Hind (LH) quarters, both yielded a CMT score of 2+ (distinct gel formation/thickening), whereas the remaining quarters were negative. The collected milk exhibited a normal gross physical appearance with no visible clots, flakes, or discoloration. Milk samples from all four quarters were pooled for further analysis. Parallel bacteriological culturing yielded no bacterial growth.

All fungal culturing procedures were conducted strictly inside a Class II Biosafety Cabinet (BSC) to prevent environmental contamination. DNA was extracted from the pooled milk sample, including extraction blanks. PCR amplification of fungal DNA extracted from milk gave an amplicon of approximately 600 bp, NTC using NFW was run in parallel to confirm the absence of PCR reagent contamination. The sample was subsequently cultured on SDA. A single fungal growth was observed after 5 days of incubation, and no other fungal growth was detected on the media. A pure colony of the isolate, designated DAH1043FM, was successfully recovered for further antifungal susceptibility and whole-genome analysis.

### Molecular identification of DAH1043FM

The Cytation 5 multimode reader's spectrophotometric analysis showed a high-purity DNA concentration of 76.304 ng/μL (A_260_/A_280_ = 1.845; A_260_/A_230_ = 1.882). The concentration of 76.5 ng/μL of intact dsDNA was confirmed by fluorometric measurement using the Qubit 4 Fluorometer. A single, clear high-molecular-weight band without visible degradation or RNA contamination was found through qualitative analysis using 0.8% agarose gel electrophoresis. The fungal isolate, DAH1043FM, was identified using ITS gene sequencing and showed a query length of 564 bp with 99.82% identity with the reference strain *A. fumigatus* ATCC 1022 (NR_121481.1), with a query coverage of 100%. The taxonomy of this fungus is Eukaryota > Fungi > Dikarya > Ascomycota > Pezizomycotina > Eurotiomycetes > Eurotiomycetidae > Eurotiales > Aspergillaceae > Aspergillus > Fumigati > *Aspergillus fumigatus*. The sequence of the ITS gene of *A. fumigatus* DAH1043FM was submitted to the NCBI GenBank database, and an accession number (OZ489555.1) was obtained. A phylogenetic tree was constructed using the maximum likelihood method in MEGA v.12 and further visualized in iTOL, showing the species-level identity. The strain DAH1043FM forms a tight, highly supported monophyletic clade with *A. fumigatus* NR_121481.1 (bootstrap value: 100%), distinctly separating it from closely related *Aspergillus* species such as *A. fischeri* and *A. lentulus* (Figure 1).

**Figure 1:**
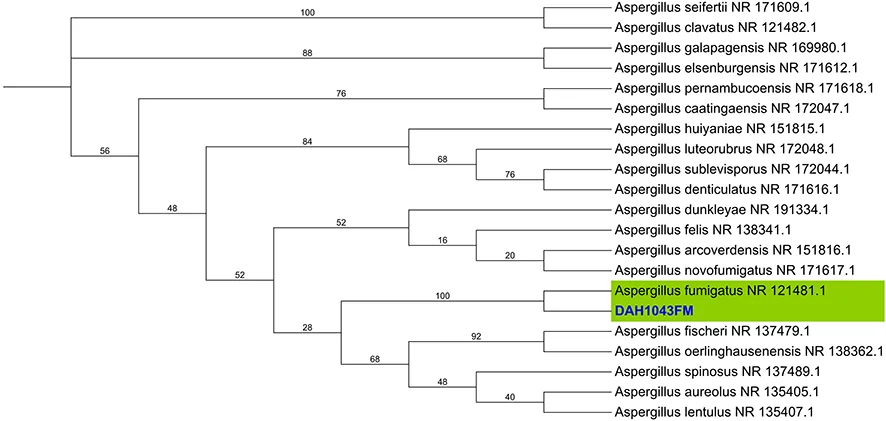
Phylogenetic analysis of *Aspergillus fumigatus* DAH1043FM.

### Antifungal susceptibility testing

The antifungal susceptibility pattern of *A. fumigatus* DAH1043FM was determined against eight different antifungal drugs. Amphotericin B (polyene); anidulafungin, micafungin, and caspofungin (echinocandins); and fluconazole, itraconazole, posaconazole, and voriconazole (azoles) were among the drugs. The ICMR breakpoints were used to interpret and compare the MIC values (Center of Expertise in Mycology Radboudumc/CWZ, 2019).

The isolate was found to be susceptible to amphotericin B (0.5 μg/mL), itraconazole (0.5 μg/mL), posaconazole (0.12 μg/mL), and voriconazole (0.12 μg/mL), and intrinsically resistant to fluconazole (128 μg/mL) (Hosseini et al., 2024). MIC breakpoints for anidulafungin, caspofungin, and micafungin are not currently available, but the high MIC value (>8 μg/mL) indicates off-scale values (Table 1).

**Table 1:** Antifungal sensitivity pattern of *Aspergillus fumigatus* DAH1043FM.

Organism	Antifungal drugs	MIC value (μg/mL)
*Aspergillus fumigatus* DAH1043FM	Amphotericin B	0.5
	Anidulafungin	>8
	Caspofungin	>8
	Micafungin	>8
	Fluconazole	128
	Itraconazole	0.5
	Posaconazole	0.12
	Voriconazole	0.12

### Genome characterization

A total of 235 contigs larger than 500 bp were identified in the initial genome assembly, with an N50 value of 257,687 bp. Of these, 197 contigs were shorter than the N50 value and were assessed for redundancy during assembly curation. One duplicated contig of 11,680 bp with 99% sequence identity to another contig was identified and subsequently removed. Following removal of this redundant contig, the final curated genome assembly consisted of 234 non-redundant contigs. The circular genome map shows the distribution of genomic features, including GC content, GC skew, coding sequences, transcripts, repetitive regions, and predicted secondary metabolite BGCs across the genome of *A. fumigatus* DAH1043FM (Figure 2).

**Figure 2:**
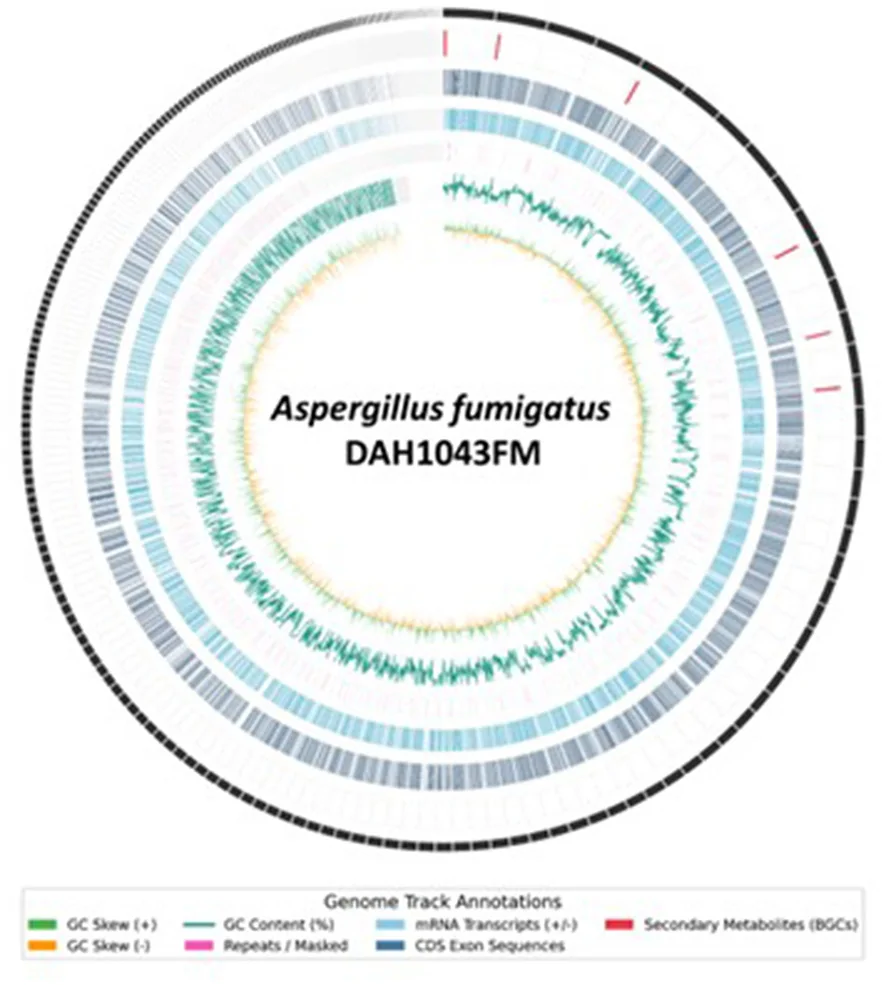
Circular genomic map of *Aspergillus fumigatus* DAH1043FM. Concentric tracks are displayed from the outermost ring to the innermost ring: (1) Outermost track: Assembled genome contigs (black blocks). (2) Track 2 (Red): Predicted secondary metabolite BGCs. (3) Track 3 (Dark Blue): Coding sequences (CDS/exon sequences). (4) Track 4 (Light Blue): Predicted mRNA transcripts (+/- strands). (5) Track 5 (Pink): Repetitive and masked genomic regions. (6) Track 6 (Teal Line): Percentage GC content plot. (7) Innermost Track (Green/Orange): GC skew analysis [green = positive GC skew (+), orange = negative GC skew (-)].

Header standardization, contig sorting, and renaming were performed for downstream studies without changing nucleotide sequence, assembly size, or contig number. Repeat analysis yielded 723,898 bp of repeated sequence, which makes up 2.36% of the entire genome. The final soft-masked assembly consisted of 234 scaffolds with a total length of 30,692,779 bp. The low repetition content demonstrated a high-quality assembly for subsequent structural and functional genome annotation (Table 2).

**Table 2:** General features of the *A. fumigatus* DAH1043FM genome.

Category	Parameters	Value
Raw sequencing data	Total raw reads (sequences)	804,946
	Total raw bases	1,942,835,025 bp (~1.94 Gb)
	Mean read length	2,413.6 bp
	Median read length	1,443.0 bp
	Read length N50	4,047.0 bp
	Maximum read length	76,604 bp
	Mean read quality	16.4
	Good quality reads (≥ Q20)	2,09,743 (26.1%)
	Good quality bases (≥ Q20)	417,046,000 bp (~417.0 Mb)
	Estimated raw sequencing depth	63.3x
Assembly statistics	Initial contigs (≥ 500 bp)	235
	Total length	30,704,459 bp (~30.7 Mb)
	Largest contig	6,95,147 bp
	GC (%)	49.5
	N50	2,56,687 bp
	N90	69,706 bp
	L50	38
	L90	133
Genome completeness	Complete BUSCOs (C)	95.50%
	Complete and single-copy BUSCOs (S)	91.70%
	Complete and duplicated BUSCOs (D)	3.80%
	Fragmented BUSCOs (F)	0.80%
	Missing BUSCOs (M)	3.70%

### Gene prediction

A total of 3,722 high-confidence protein alignments to the genome assembly were obtained by the structural annotation pipeline using protein homology evidence from 561,666 reference proteins. A total of 1,199 conserved fungal genes were identified using BUSCO analysis and were further used to train *ab initio* gene prediction algorithms. Genome annotation completeness was assessed using BUSCO v2.0 (integrated via funannotate) against the dikarya_odb9 lineage dataset, evaluating 1,312 expected ortholog groups (achieving 94.9% complete BUSCOs: 91.4% single-copy, 3.5% duplicated).

*Ab initio* gene prediction using Augustus, SNAP, and Glimmer HMM revealed 9,169, 9,943, and 10,776 gene models, respectively. Integration of these predictions using EvidenceModeler generated 10,103 consensus gene models. EvidenceModeler weights were set to standard funannotate defaults such as 10 for transcript evidence, 1 for protein alignments, and 1 for *ab initio* gene predictions. Further quality filtering removed 123 low-confidence predictions, including one short gene model and 122 transposable element-associated models, which finally resulted in 9,980 predicted protein-coding genes. Additionally, 214 non-overlapping tRNA genes were identified. The final structural annotation included 10,194 gene models, including both protein-coding and tRNA genes (Table 3).

**Table 3:** Structural genome annotation statistics.

Annotation stage	Evaluation matrix	Value
Input assembly baseline	Final non-redundant contigs	234
	Total genome length	30,692,779 bp
	Repetitive sequences masked	2.36%
*Ab initio* predictors	Augustus predictions	9,169
	Augustus High-Quality (HiQ)	460
	SNAP predictions	9,943
	GlimmerHMM predictions	10,776
Evidence blending (EVM)	Total input models to EVM	30,263
	Initial consensus EVM models	10,103
Post-prediction filtering	Models removed (total)	123
	—* Short models (< 50 aa)*	*1*
	—* Transposable elements*	*122*
	Remaining protein-coding genes	9,980
Non-coding RNA	tRNAscan models	214
Final structural annotation	Total gene models	10,194

### Functional annotation

Functional annotations were derived by querying UniProt and Pfam databases using an E-value cutoff threshold of ≤ 1 X 10^−5^. Functional annotation of 10,194 predicted gene models was performed using multiple complementary databases and annotation resources. A total of 12,846 conserved domain annotations were identified using protein domain analysis against the Pfam database. A total of 1,044 high-confidence gene and protein functional classifications were generated by similarity searches against the UniProt database.

Orthology-based annotation using EggNOG-mapper revealed 20,401 COG and EggNOG functional annotations, enabling classification of genes into conserved functional categories. Gene names and product descriptions were given to 3,802 gene models through integration of UniProt and EggNOG findings. Furthermore, 491 CAZyme annotations were found using dbCAN, and 303 protease-related annotations were found using the MEROPS database. BUSCO-related functional characterization further assigned 1,263 conserved dikarya ortholog annotations.

Because individual genes could receive multiple annotations from different databases, these annotation categories were not mutually exclusive. During integration of the annotation results, Funannotate identified 25,917 duplicated annotations and retained 16,991 valid, non-redundant annotations in the final annotation dataset. These integrated annotations provided a comprehensive functional characterization of the predicted gene set (Table 4).

**Table 4:** Summary of functional annotation.

Functional database	Annotations found
Pfam	12,846
UniProt	1,044
EggNOG	20,401
Gene2Product	3,802
MEROPS	303
CAZymes annotation (dbCAN)	491
BUSCO (dikarya annotation)	1,263
Final valid non-redundant annotations after integration	16,991

### Genome-based confirmation of *A. fumigatus*

The isolate DAH1043FM showed 99.72% average nucleotide identity with the reference genome of *Aspergillus fumigatus* Af293, confirming its species identity as *A. fumigatus*. The raw ONT sequencing reads have been deposited in NCBI under BioProject accession number PRJEB104476 and BioSample accession number SAMEA122955174, with the raw-read accession number ERR17730998. The assembled genome has been submitted under accession number GCA_988205085.

### Mutational analysis

Comparative sequencing analysis of antifungal targets, ergosterol biosynthesis, and transporter-associated genes identified numerous SNPs, insertions/deletions (indels), truncations, and terminal elongation events in strain DAH1043FM. The *erg1* gene in the ergosterol biosynthesis pathway has a unique A154V substitution, which causes valine to replace alanine at position 154; the *erg3B* gene has an M99L substitution; and *erg12* has an A107S amino acid alteration. The *erg5* gene exhibited a different C-terminal elongation in which an 11-amino acid extension (DDCHLTFRPRA) took the place of the terminal valine (V482), which increases the protein length from 482 to 492 amino acids. The last 12 amino acids (LYSRKFGNEELM) found in reference strains Af293 and A1163 were lost in the premature C-terminal truncation of *erg7A*. In contrast, *erg7B* exhibited both an internal deletion and a terminal extension, including a 16-amino acid deletion (VSDCISEALKAVIVLQ) spanning residues 441–456, a missense substitution (Y496S), and a frameshift-associated replacement of the canonical terminal hexapeptide (KYPHLV) with a unique extended tail sequence (NTPTSCRRWATMDVSIKQ), resulting in a total protein length of 716 amino acids. The *erg8* protein contained a complex internal indel region consisting of a 9-amino acid deletion (RGMQMVLCD), substitution of the sequence VECGSQTPSMVRKVLE with AECKWSSVTSNV, and an adjacent 4-amino acid deletion (WRKQ), while both N- and C-terminal regions remained intact.

Additional mutations were observed in other genes associated with membrane biosynthesis and cellular function. The *fen1* gene retained an intact open reading frame but displayed complete replacement of residues 5–39 by an alternative 35-amino acid sequence (HLFQVIQENAPDAIKAGDIKNHFGRKVAILTSCRS-MS), after which sequence identity returned to that of the reference strains. The *hmg2* protein contained three missense substitutions (R86H, L219F, and Y397C), a 9-amino acid deletion (VRPSFFLDP) spanning residues 107–115, and a prominent C-terminal elongation beginning at residue 1,046, resulting in an expanded protein of 1,121 amino acids. A full-length open reading frame with a single conservative P22L mutation near the N-terminus was found in the DNA polymerase epsilon catalytic subunit gene (*pol2*). Additionally, variations were found in *top2* and *vma16*. Specifically, *top2* exhibited a 74-amino acid N-terminal extension and two missense substitutions (K1257N and Y1403D), and *vma16* exhibited a 74-amino acid N-terminal extension upstream of its core proteolipid domain without these substitutions.

Several transporter-associated genes exhibited sequence polymorphisms. The glucose transporter *mfs1* contained a localized 20-amino acid hyper variable substitution region between residues 30 and 50 while remaining otherwise conserved. The ABC multidrug transporter *mdr1_1* retained full-length integrity but harbored three amino acid substitutions, E13K, G23C, and V299I. Comparative analysis of the *mdr4* locus identified *mdr4_4* as the most complete gene and revealed four N-terminal substitutions (N3K, T4V, S61T, and T62R) together with the resolution of previously unannotated sequence loops.

Read-level alignment verification across all target loci confirmed 100% sequence concordance (QUAL > 30, DP > 45–68 X) between mapped reads and the consensus assembly, establishing these mutations as true genomic features. All mutations are listed in Table 5. Collectively, these results show significant genetic variation within DAH1043FM, including frameshift-associated elongations, indels, truncations, and SNPs in genes associated with drug transport, cellular maintenance, and ergosterol production.

**Table 5:** Mutational profiling and structural modifications identified in targeted genes.

Gene	Mutation	Observation
*erg12*	A107S	Single mutation (SNP)
*erg1*	A154V	Single mutation (SNP)
*erg3B*	M99L	Single mutation (SNP)
*erg5*	C-terminal Extension	11-amino acid C-terminal tail extension (DDCHLTFRPRA) replacing terminal Valine
*erg7A_1*	C-terminal Truncation	Premature termination with a frameshift; lacks the final 12 residues (LYSRKFGNEELM) and ends in PL
*erg7B*	Complex Structural Mutation	16-amino acid internal deletion (VSDCISEALKAVIVLQ), Y496S substitution, and a 14-amino acid C-terminal tail extension (NTPTSCRRWATMDVSIKQ)
*erg8*	Complex Internal Indel	Localized 9-amino acid deletion (RGMQMVLCD), multiresidue replacement (VECGSQTPSMVRKVLEAECKWSSVTSNV), and an adjacent 4-amino acid deletion (WRKQ)
*pol2*	P22L	Single mutation (SNP)
*fen1*	N-terminal Disruption	Localized hypervariable block substitution spanning residues 5 to 39 (HLFQVIQENAPDAIKAGDIKNHFGRKVAILTSCR-SMS)
*hmg2*	Complex Mutation	Three single mutations (R86H, L219F, Y397C), an internal 9-AA regulatory deletion (VRPSFFLDP), and a major 76-AA C-terminal frameshift elongation
*top2*	N-terminal Extension + SNPs	74-amino acid N-terminal cap elongation accompanied by two downstream point mutations (K1257N and Y1403D)
*vma16*	N-terminal Extension	74-amino acid upstream unannotated framework extension prior to the core proteolipid domain
*mfs1*	N-terminal Variation	Highly localized 20-amino acid hypervariable patch substitution spanning residues 30 to 50
*mfs2_1*	Incomplete Sequence	Sequence fragment displaying extensive5′ gaps and terminal deletion (likely an assembly or sequencing artifact)
*mdr1_1*	E13K, G23C, V299I	Three independent single mutations (SNPs) across the transporter reading frame
*mdr4_4*	N3K, T4V, S61T, T62R	Four N-terminal single mutations (SNPs) paired with successfully resolved unannotated reference sequence loops

### Gene clusters involved in the biosynthesis of secondary metabolites

AntiSMASH predicted 36 potential BGCs in the genome of *A. fumigatus* DAH1043FM. Nine Type-1 polyketide synthase (T1PKS), seven non-ribosomal peptide synthetase (NRPS), eight NRPS-like clusters, five terpene clusters, one indole-containing cluster, one betalactone, and five hybrid clusters, including T1PKS-indole, T1PKS-NRPS, T1PKS-NRPS-indole, and NRPS-indole hybrids, were among the predicted clusters (Figure 3).

**Figure 3:**
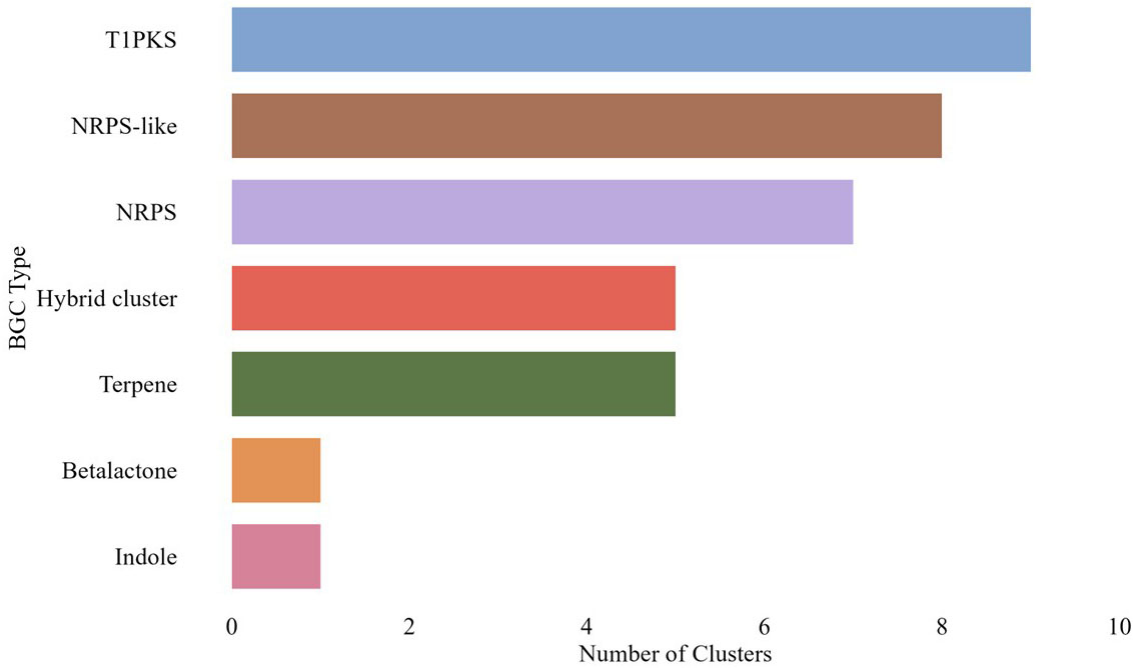
Distribution of predicted BGCs in the genome of *A. fumigatus* DAH1043FM.

Several secondary metabolite BGCs in strain DAH1043FM showed high levels of similarity to previously identified fungal metabolite pathways. For comparative interpretation, BGCs showing ≥70% similarity to characterized secondary metabolite biosynthetic pathways were considered. The BGCs for fumiquinazoline (region 95.1), clavaric acid (regions 34.1 and 82.1), and naphthopyrone (region 56.1) showed the greatest similarity (100%), while regions 35.1 and 121.1 showed 80% similarity to fumagillin/fumagillol and neosartoricin B BGCs, respectively. Region 7.1 showed 84% similarity to the gliotoxin BGCs, a significant virulence-associated metabolite pathway in *A. fumigatus*, and trypacidin showed 71% similarity in region 220.1. Overall, these results show that DAH1043FM has a wide variety of predicted gene clusters involved in the biosynthesis of secondary metabolites, including several highly conserved pathways associated with virulence, mycotoxin formation, and other bioactive secondary metabolites (Table 6). The predicted BGCs exhibited varying levels of similarity to characterized secondary metabolite pathways and showed substantial variation in cluster size (Figure 4).

**Table 6:** Potential secondary metabolites of *A. fumigatus* DAH1043FM.

Region	BGC Type	Predicted Metabolite/Cluster	Class	Similarity
7.1	NRPS, NRPS-like	Gliotoxin/Bisdethiobis(methylthio)gliotoxin	NRP	84%
34.1	Terpene	Clavaric acid	Terpene	100%
35.1	T1PKS, NRPS, Indole	Fumagillin/β-trans-bergamotene/Fumagillol	Terpene + Polyketide	80%
56.1	T1PKS	Naphthopyrone	Polyketide	100%
82.1	Terpene	Clavaric acid	Terpene	100%
95.1	NRPS	Fumiquinazoline A/C/D	NRP	100%
121.1	T1PKS, Indole	Neosartoricin B	Polyketide	80%
220.1	T1PKS	Trypacidin	Polyketide	71%

**Figure 4:**
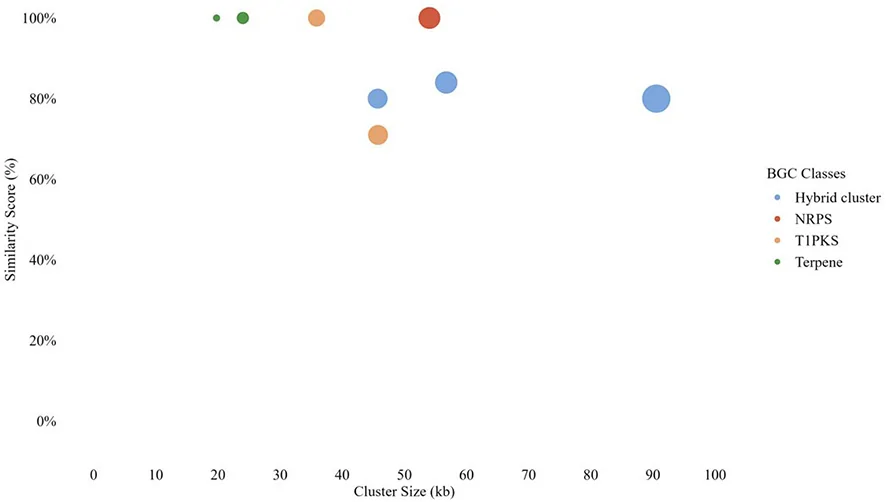
Distribution of predicted BGCs based on cluster size and similarity to characterized secondary metabolite pathways.

## Discussion

Mastitis is one of the most common and significant diseases affecting dairy animals worldwide (Krishnamoorthy et al., 2021). It is characterized by inflammation of the mammary gland, primarily caused by bacterial and fungal pathogens that invade the udder tissue (Sharun et al., 2021). The isolation of fungal pathogens from bovine mastitis cases has been reported in recent years, suggesting that fungal intramammary infections are growing rapidly (Ksouri et al., 2014). Antibiotics are still the primary treatment for bovine mastitis, but their indiscriminate and prolonged use has led to the development of antimicrobial resistance, which lowers the effectiveness of treatment against pathogenic microorganisms (Che et al., 2023).

This study is the first to characterize a filamentous fungus associated with mastitis in bovine milk using WGS. This is significant because fungal mastitis genomics has remained relatively limited, whereas genome-wide mastitis research has grown mainly in bacterial pathogens. Notably, the most recent bovine example is a 2024 whole-genome study of *Candida tropicalis*, not a filamentous mold (Jiang et al., 2024). In our study, we isolated the fungus from the milk of a bovine with mastitis and identified it using ITS region sequencing. This fungus is an opportunistic filamentous mold that causes mastitis in well-reported cases (Ksouri et al., 2014; Morales-Ubaldo et al., 2023; Ahmed et al., 2024). At present, research on *A. fumigatus* is primarily focused on human diseases, and research on bovine mastitis is rare. Furthermore, most work describes clinical risks and therapeutic resistance, with little research on the genome. Therefore, understanding the genomic characterization of *A. fumigatus* was of great importance for our study of bovine mycotic mastitis.

The antifungal profile of strain DAH1043FM is biologically convincing in a number of ways and internally consistent. Amphotericin B, itraconazole, voriconazole, and posaconazole all had low MICs against the isolate, with off-scale MIC values for echinocandins, although fluconazole showed high MIC activity against *A. fumigatus*. Most significantly, neither *cyp51A* nor *cyp51B* showed any conventional resistance-associated mutations, indicating that the triazole phenotype is consistent with the genotypic evidence. Since promoter tandem repeats and hotspot substitutions affecting *cyp51A* remain the most extensively validated resistance mechanisms in this species, isolates lacking such changes typically remain within the wild-type susceptibility range (Toyotome et al., 2021; Kühbacher et al., 2023). This is precisely the pattern expected for a wild-type azole-susceptible *A. fumigatus* isolate.

However, recent studies have demonstrated that *A. fumigatus*'s azole sensitivity cannot be entirely caused by *cyp51A*. Additional azole-tolerance pathways discovered through experimental azole exposure and pan-azole resistance connected to *cox7c* disruption are examples of unconventional mechanisms involving mitochondrial function, stress adaptation, altered intracellular drug accumulation, and broader regulatory changes (Toyotome et al., 2021; Chen et al., 2022).

Although there were no significant hotspot alterations in the *fks* gene, DAH1043FM showed off-scale MIC values to anidulafungin, caspofungin, and micafungin (echinocandins). Target-site modification can directly induce resistance, as revealed by the *fks1* hotspot mutation in *A. fumigatus* (Jiménez-Ortigosa et al., 2017). However, mutations are not always the cause of *A. fumigatus*'s decreased susceptibility to echinocandins. Increased *fks* expression was suggested as a contributing mechanism in an earlier clinical study that showed a caspofungin reduced-susceptibility phenotype in the absence of an *fks1* mutation (Arendrup et al., 2008).

Even when the *fks* gene is still wild-type, this phenotype can be explained by many physiologically reasonable hypotheses. The cell wall integrity network, compensatory chitin production, and stress-response circuitry in *A. fumigatus* are closely linked to the echinocandin response. Research has demonstrated that calcineurin-dependent regulation of chitin synthesis plays a role in caspofungin tolerance and paradoxical growth. This suggests that cell wall composition remodeling can mitigate the impact of β-1,3-glucan synthase inhibition without necessitating structural modification of the target enzyme (Fortwendel et al., 2010). Similarly, azole and echinocandin susceptibility is modulated by Hsp90-dependent stress responses, and *A. fumigatus* is more susceptible to caspofungin when Hsp90 function is disrupted (Lamoth et al., 2014). When combined, these findings support the theory that, rather than being solely caused by a traditional *fks* mutation, the off-scale echinocandin MICs in DAH1043FM most likely represent a tolerance-like or reduced-susceptibility state driven by regulatory adaptation, cell wall salvage responses, or altered glucan-synthase pathway expression.

Therefore, it is more appropriate to conclude that DAH1043FM showed off-scale MIC values for the echinocandins. Since the interpretation criteria for molds are still lacking, this distinction is particularly crucial. There are fewer species-drug-specific clinical breakpoints available for *Aspergillus* species than for common yeasts, and epidemiological cutoff values are frequently more informative than strict susceptible/resistant labels (Espinel-Ingroff et al., 2011). Therefore, the echinocandin results should be interpreted together with the genomic data and the known stress adaptation mechanisms of *A. fumigatus*. This approach provides a more accurate interpretation of antifungal susceptibility.

The current investigation also focuses on genome-based prediction of secondary metabolic potential. The presence of predicted BGCs for gliotoxin, fumiquinazoline, fumagillin, and trypacidin reflects the secondary metabolic framework of isolate DAH1043FM, though functional production of these metabolites remains to be evaluated. Gliotoxin is the most strongly supported virulence-associated metabolite in *A. fumigatus*. According to recent research, gliotoxin reduces phagocytic competence, attenuates immune-cell recruitment, and impairs macrophage lipid-mediator responses to inhibit innate immune activity (Günther et al., 2024). Such an action is very relevant in the context of bovine mastitis since successful intramammary colonization requires escape of epithelial defenses and persistence despite clearance by neutrophils and macrophages. Therefore, the prediction of a gliotoxin-like cluster in DAH1043FM highlights its genomic biosynthetic potential, underscoring the need for future functional studies to determine its role in host interactions.

The predicted BGCs for trypacidin, fumagillin, and fumiquinazoline further support this interpretation. These metabolites are part of the known *A. fumigatus* secondary metabolite arsenal linked to host interaction, cytotoxicity, and niche fitness, even if their precise role in bovine intramammary infection has not yet been functionally confirmed *in vivo*. The predicted BGCs for gliotoxin (84% similarity), fumiquinazoline (100%), fumagillin (80%), and trypacidin (71%) highlight the secondary metabolic potential of *A. fumigatus* DAH1043FM. In characterized strains, these metabolic pathways are linked to host interactions and environmental fitness; however, their functional expression and specific contribution to bovine intramammary infection remain to be established through future *in vivo* studies.

The current strain is not similar to the well-known triazole-resistant genotypes that are dominated by hotspot alterations or tandem repetitions of the *cyp51A* promoter (Kühbacher et al., 2023). Additionally, it does not fit the more uncommon but traditional theory of echinocandin resistance caused by *fks1* hotspot mutations (Jiménez-Ortigosa et al., 2017). The off-scale values for anidulafungin, caspofungin, and micafungin do not appear to result from the conventional resistance mechanism involving target-site mutations. Instead, it is likely associated with broader cellular adaptation mechanisms that enable the fungus to tolerate drug-induced stress, while azole susceptibility is substantially genotype-concordant. That profile is entirely compatible with understanding of *A. fumigatus* resistance biology, in which not all clinically relevant phenotypes are explained by the usual marker genes (Toyotome et al., 2021; Chen et al., 2022).

Overall, this study provides a genomic and phenotypic characterization of *Aspergillus fumigatus* strain DAH1043FM isolated from bovine milk. Whole-genome sequencing identified predicted BGCs for secondary metabolites, a wild-type azole susceptibility profile, and off-scale echinocandin values (>8 μg/mL). These findings demonstrate the utility of WGS for baseline genomic surveillance of bovine intramammary fungal isolates. Combining these results supports the conclusion that WGS is crucial for understanding the molecular basis of persistence, therapeutic challenge, and secondary metabolite potential in mastitis-associated *A. fumigatus*, rather than just a confirmatory method for fungal identification in bovine mastitis (Günther et al., 2024; Jiang et al., 2024).

## Limitation

A primary limitation of this study is that it evaluated a single bovine mastitis-derived isolate. While this provides valuable baseline genomic insights into filamentous fungal mastitis, findings from a single strain cannot be broadly generalized across all mastitis-associated *A. fumigatus* populations. Additionally, the present study relied on genome-based prediction of virulence factors and antifungal resistance determinants. Comprehensive pathogen–host interaction analyses and experimental validation of candidate genes were beyond the scope of this study and require further investigation to better understand the molecular mechanisms underlying the pathogenicity of *A. fumigatus* in bovine mastitis. In addition, the expression levels of virulence and resistance-associated genes were not evaluated. Therefore, future studies involving transcriptomic analyses and gene expression profiling are needed to determine the functional significance of the identified genes and their potential role in pathogenicity and antifungal response.

## Conclusion

In summary, we isolated and characterized a strain of *A. fumigatus* DAH1043FM from a cow with mastitis. Experimental results show that *A. fumigatus* possesses predicted BGCs and is sensitive to azoles, showed off-scale values for echinocandins, and was intrinsically resistant to fluconazole. We identified the drug resistance genes using genome sequence analysis and conducted a comparative genomics investigation using the reference genome of *A. fumigatus*. Additionally, genome mining identified BGCs associated with the biosynthetic potential for several secondary metabolites, including virulence-associated metabolites; however, their actual production and contribution to pathogenicity were not experimentally validated. This is the first genomic study on a filamentous fungus isolated from a bovine animal suffering from mastitis. We also highlight the necessity of putting action plans into place to fight antifungal resistance and encourage the prudent use of antifungal medications.

## Future scope

Apart from *A. fumigatus*, there are various fungi recovered from milk of bovine mycotic mastitis; therefore, more comprehensive work needs to be done, especially on *Candida* species, which are frequently reported in fungal mastitis. WGS of more fungal isolates from various geographical locations would help in identifying lineage-specific adaptations, virulence factors, and resistance mechanisms responsible for mammary infections, since the genomic investigations of mastitis-associated fungi are still limited. The resistance and virulence genes identified through WGS should be experimentally validated using gene knockout, overexpression, or transcriptomic approaches to determine its precise role in pathogenicity and antifungal tolerance.

## Data Availability

The datasets presented in this study can be found in online repositories. The names of the repository/repositories and accession number(s) can be found in the article/Supplementary material.
