# Supplementary material for: Antifungal susceptibility and genomic profile associated with biosynthetic gene clusters of bovine mastitis-derived Aspergillus fumigatus DAH1043FM
Source: Front Microbiol. 2026 Sep 30;17:1928624. doi: 10.3389/fmicb.2026.1928624 (PMC13661572; doi:10.3389/fmicb.2026.1928624)
Supplement: Supplementary file 1 [file Supplementary_file_1.pdf]

Supplementary Table 1: MIC breakpoints and interpretive criteria used for antifungal susceptibility testing of *A. fumigatus*.

Antimicrobial Agent	MIC (μg/mL)			Reference
	S	I	R	
Amphotericin B	≤ 2	NA	≥ 4	ICMR, 2019
Itraconazole	≤ 1	NA	≥ 2	ICMR, 2019
Posaconazole	≤ 0.5	NA	≥ 1	ICMR, 2019
Voriconazole	≤ 1	NA	≥ 2	ICMR, 2019
Fluconazole	Intrinsic resistance			Hosseini et al., 2024
Caspofungin	No established breakpoints			NA
Micafungin	No established breakpoints			NA
Anidulafungin	No established breakpoints			NA
